# What May Our Patients Reasonably Expect Shall Be Accomplished by Operations upon the Teeth

**Published:** 1875-06

**Authors:** Geo. H. Cushing

**Affiliations:** Chicago.


### ARTICLE IV.

### *What may our Patients Reasonably Expect shall be Accomplished by Operations upon the Teeth.*

BY GEO. H. CUSHING, D. D. S., CHICAGO.

Read before the New York Odontological Society.

The question which it is the purpose of this paper briefly to consider is one which very largely affects the interests of the dental profession. If the true solution of it could be clearly arrived at and accepted by the profession, their interests could not fail to be favorably affected thereby; while so long as it remains an unsettled question, or is but imperfectly understood, or carelessly defined, it must prove more or less detrimental to professional interests, and, it may also be added, to the interests of humanity, for the two are identical.

It is quite certain that this subject has not engaged the serious thought of the profession to the extent that its importance demands; and the endeavor will be made in this paper to so answer the inquiry in a general way, as to arouse attention to its importance, with the hope that through your discussions and the influence of your Society it may be brought more generally before the mind of the profession at large, with such an impress of the best thought upon it from your members as will secure its proper consideration, and lead to its speedy and correct interpretation.

The true professional mind could hardly rest satisfied without being able to give an unequivocal reply to this query, and yet it is more than probable that the diversity of answers would be almost as great as the number of individuals replying.

The general public, who are especially interested in the truthful reply to this question, can have no proper appreciation of the value of our services until this question can be answered truthfully and with a common understanding by the profession at large; and they have a right to demand that it shall be so answered.

It will not be undertaken here to discuss the question as to what the public may have a right to expect from the assumed or from the apparent attitude of the profession toward them.

Whatever that attitude is—whether true or false—we shall not here attempt to say. If it be false, it must, as speedily as possible, be corrected; if true, it will need no reference except as will naturally come from a consideration of the answer to the question under consideration.

It is not at all the purpose of this paper to attempt an exhaustive essay. It will only be attempted to place the subject before you in such a manner as to elicit a full discussion of all points incident and essential to a full understanding of the question. It is essential to anything like a fair consideration of this question, that dental practitioners should be properly classified. Of course this can only be done in a general sense, as it might be said that there are as many classes as individuals.

For the purpose of this paper, it will be proper to class them under two heads: one class embracing all those who are endeavoring constantly, honestly and conscientiously to do the very best that can be done, and who avail themselves of the various means to that end which are within their reach, *i. e.*, association, free interchange of views and experiences, and the literature of the profession, both standard and periodical. The other class, of course, embraces all those who do not avail themselves of these means, and who, either from prejudice, ignorance or dishonesty, choose to remain in the disreputable rear-guard of a truly noble profession. The importance of this distinction needs not to be argued, and in treating the subject of this paper, the first mentioned class of practitioners will be understood as the profession—the authors of the operations under consideration.

In attempting to answer the query started by this paper, very many points require consideration. A direct answer, and perhaps one which would be given by the greater number of respondents, would be that patients might reasonably expect that their teeth would be preserved to extreme age, or until lost from other causes than caries. This might, in a certain sense, be affirmed with entire truth, and yet such results are generally so contingent upon conditions beyond the control of the dentist, that it would not be right to say that such expectation would be reasonable without stating the conditions on which alone it is possible, in many cases, for this statement to be true.

The conditions of course are, that patients shall obey implicitly the necessary instructions of their professional advisers.

Now, just here it is proper to call your attention to a very grave error which patients are apt to cherish, and one which, unfortunately, too many of the profession are inclined to foster, and that is, the supposition that teeth once well filled will last forever. You are all doubtless aware to what extent this false idea prevails, and you know as well how in part it has come to prevail—because some practitioners not only have allowed their patient to take this for granted, but have many times assured them that operations would last indefinitely—never requiring renewal. Not only do a large number of persons hold this view regarding operations performed, but very many of them seem to think that, having the teeth once put in good condition, they will never require anything more to be done, either to those already operated upon or to any others—as though there was some conservative property residing in the gold which is placed in certain teeth that would surround with its saving influence all the teeth in the head.

Until the public mind is disabused of this idea, and the profession generally make it a rule to instruct their patients that these operations are, in the very nature of things, liable to be but temporary in their character, we can have no basis upon which patients can found any reasonable expectations. Again, there are some individuals who have no faith at all in the operation of filling teeth, because they have found in their own experience, or in that of their friends, that certain teeth that had once been filled were, in a few years afterwards, hopelessly decayed.

This conviction is arrived at on their part in most cases because of the above named false promise that teeth once filled require no further thought, and consequently they have neglected the essential after-treatment and professional supervision necessary to insure the best results.

These points are emphasized here because they enter so largely into the question which it is sought to solve, and because it is certain that the false position which the profession to-day occupies before the public (to whatever extent it is false) is due mainly to a lack of a proper appreciation of its duties in this regard.

Probably no one would deny that, if children were placed under the dentist's hands at the proper age, and he was allowed to have entire control of them, so far as their teeth were concerned, until maturity, their teeth could all be preserved up to that time; and yet how many parents are there who think that their children *are* thus placed in the dentist's hands, and that they are really complying with all his reasonable demands, who yet find that results of operations are continually disappointing them, and who are not made to realize that they have criminally neglected the instructions of the dentist. How many are there who have been instructed by their dentist to bring their children to him every three months, who bring them only every one, two or three years. And again, how many have been instructed in the importance of a thorough cleansing of the teeth, especially after eating, who think their children do all that is necessary by brushing their teeth upon rising and retiring, neglecting entirely the only periods when brushing can be said to be of any great value? Thus, while children may be nominally in the hands of the dentist, yet they are in fact not at all so in the proper sense, and a failure to arrive at the best results is the consequence; a failure which is oftentimes unjustly charged to the dentist. This laxity on the part of patients and parents is partly the fault of the profession, in that they do not sufficiently impress upon them the importance of observing their instructions; but it is not altogether so, for there are many who will not follow such instructions, however carefully given.

It cannot be too forcibly urged that the profession could do very much towards insuring compliance with their instructions than they are doing, if they were themselves thoroughly impressed with the importance of the subject.

As in the cases supposed above, the circumstances attending them would be essential elements in making a prognosis regarding them, so, in general (with every case having some individual peculiarity probably,) must be considered the different circumstances under which operations are made; the various characteristics of the individual mouths which are treated; the great liability to decay in the teeth of some individuals, and the less liability in those of others; all these considerations must have weight in determining the solution of this question.

The fact cannot be ignored, that the mere operation of filling teeth does not alter their character as organs liable to disease—the same after the operation as before—that the operation does not remove the causes of decay, which may still continue active, or become so at some future time, and which will again prove destructive of the other parts of the teeth under conditions favorable to such destruction. Were it otherwise, a very positive affirmation could be made of the benefits which might be expected from these operations.

In view of the premises thus set forth, what then may be offered as a proper answer to the question sought to be solved? This, viz: patients may reasonably expect from operations on the teeth just what an honorable, conscientious practitioner promises them they may expect under the conditions he (the dentist) may impose; that the benefit derived from operations will be in the precise ratio to the obedience to such instructions, and that patients, doing their duty as faithfully as the dentist does his, may hope to retain their teeth as useful organs to an indefinite period. These things may reasonably be expected; but there are some things patients may not reasonably expect.

They may not expect that so long as they are mortal will they be entirely exempt from danger of caries, nor need they think that mere manipulative skill, no matter how high an order, bestowed in making the most faultless operations, will justify them in continuous disobedience to the laws of health, or the disregard of those measures of prevention which experience has demonstrated to be essential for the preservation of the teeth, without being called upon to pay the penalty.

Keeping in view these considerations, it will be the fault of patients themselves if results *seem* to bring discredit upon the profession.

The duty of the profession in this regard is very clear—that they see to it that they do not promise more than they perform, and that they do uot allow their patients to comfort themselves with hopes not founded in reason.

